# Fluoropolymer/Glycidyl Azide Polymer (GAP) Block Copolyurethane as New Energetic Binders: Synthesis, Mechanical Properties, and Thermal Performance

**DOI:** 10.3390/polym13162706

**Published:** 2021-08-13

**Authors:** Minghui Xu, Xianming Lu, Ning Liu, Qian Zhang, Hongchang Mo, Zhongxue Ge

**Affiliations:** 1 State Key Laboratory of Fluorine & Nitrogen Chemicals, Xi’an Modern Chemistry Research Institute, Xi’an 710065, China; 2 Department of Energetic Materials Science and Technology, Xi’an Modern Chemistry Research Institute, Xi’an 710065, China; luxianming@126.com (X.L.); flackliu@163.com (N.L.); qian3545267@163.com (Q.Z.); hongchangmo@163.com (H.M.); gzx204@163.com.cn (Z.G.)

**Keywords:** energetic binder, block copolyurethane, sensitivity, mechanical property, thermal behavior

## Abstract

In order to enhance the application performance of glycidyl azide polymer (GAP) in solid propellant, an energetic copolyurethane binder, (poly[3,3-bis(2,2,2-trifluoro-ethoxymethyl)oxetane] glycol-*block*-glycidylazide polymer (PBFMO-*b*-GAP) was synthesized using poly[3,3-bis(2,2,2-trifluoro-ethoxymethyl)oxetane] glycol (PBFMO), which was prepared from cationic polymerization with GAP as the raw material and toluene diisocyanate (TDI) as the coupling agent via a prepolymer process. The molecular structure of copolyurethanes was confirmed by attenuated total reflectance-Fourier transform-infrared spectroscopy (ATR–FTIR), nuclear magnetic resonance spectrometry (NMR), and gel permeation chromatography (GPC). The impact sensitivity, mechanical performance, and thermal behavior of PBFMO-*b*-GAP were studied by drop weight test, X-ray photoelectron spectroscopic (XPS), tensile test, scanning electron microscopy (SEM), differential scanning calorimetry (DSC), and thermal gravimetric analysis (TGA), respectively. The results demonstrated that the introduction of fluoropolymers could evidently reduce the sensitivity of GAP-based polyurethane and enhance its mechanical behavior (the tensile strength up to 5.75 MPa with a breaking elongation of 1660%). Besides, PBFMO-*b*-GAP exhibited excellent resistance to thermal decomposition up to 200 °C and good compatibility with Al and cyclotetramethylene tetranitramine (HMX). The thermal performance of the PBFMO-*b*-GAP/Al complex was investigated by a cook-off test, and the results indicated that the complex has specific reaction energy. Therefore, PBFMO-*b*-GAP may serve as a promising energetic binder for future propellant formulations.

## 1. Introduction

A recent trend in the field of energetic material formulations (explosives/propellants) is to replace inert binders (viz., hydroxy terminated poly butadiene (HTPB), carboxyl terminated polybutadiene (CTPB), and hydroxyl terminated polyether (HTPE), etc.) by energetic binders, which contain energetic groups such as –N_3_ (azide), nitro (C–nitro, O–nitro (nitrate ester), N–nitro (nitramine) and difluroamine groups, to impart additional energy to the systems [1,2,3]. Among energetic polymers, glycidyl azide polymer (GAP) has been extensively studied as a polymeric binder since it was first reported in a patent in 1972 by Vandenburg [4,5,6]. This is due to its high density (1.3 g cm^−3^) with positive heat of formation of +117.2 kcal mol^−1^, low glass-transition temperature (*T*_g_ = −45 °C), good thermally stability, low detonation tendency, and high burning rate (1 cm s^−1^ at 40 atmospheres) [7,8]. Thus, it offers a unique energetic binder and plasticizer system for advanced propellants and plastic bonded explosives (PBX) to achieve a higher performance, and has become a hotspot in the field of energy materials [9]. However, traditional GAP-based binders are thermosetpolymer and usually difficult to recycle. In addition, it also suffers from highly sensitive and inferior mechanical behavior (generally, the largest tensile strength σ_m_ was less than 2 MPa; the elongation at break ε_m_ was less than 200%), which is due to its high polarity of azide groups and poor flexibility of the polymer backbone [10,11,12].

In order to overcome these difficulties and obtain a better performance, various energetic polymers have been developed in the last two decades. Energetic thermoplastic elastomers (ETPE) as high performance recyclable polymeric binders with superior mechanical properties (σ_m_: 2–5 MPa, ε_m_: 200–700%), have received widespread attention in the past decades [13]. In ETPE, the crystalline hard segments forming physical cross-linking points provide mechanical strength, while the energetic soft segments provide flexibility and energy. When it heated above the melting temperature of the hard segments, the physical crosslinks between the polymer chains disappear, allowing the ETPEs to flow like a thermoplastic and fully recyclable [14,15]. Development of novel ETPE has attracted the extensive attention of many researchers in recent years [16,17].

In the past three decades, fluoropolymers have gained considerable attention in the energetic material community (such as aerial infrared decoys, igniters, tracking flares, reactive binder systems, and solid fuel rocket propellants) as high explosive binders, owing to their high densities, low coefficients of friction, long-term chemical stabilities, and good compatibility with the main ingredients (oxidizers, metal fuels, and plasticizer) [18,19,20]. Particularly, fluoropolymer possesses strong oxidation [21,22,23], for instance, the magnesium, Teflon, and Viton system (MTV) as one of the well-known compositions used in decoys and flares. The metal/fluoropolymer compositions have a special exothermic reaction heat of 9.4 kJ g^−1^, which is higher than the control compositions based on 2,4,6-trinitrotoluene (TNT) (3.72 kJ g^−1^) and cyclotrimethylenetrinitramine (RDX) (6.569 kJ g^−1^) [24]. 

In this study, fluoropolymer/GAP block copolyurethane binders were synthesized via a prepolymer process by coupling together poly[3,3-bis(2,2,2-trifluoroethoxymethyl) oxetane] glycol (PBFMO) and GAP to decrease the sensitivity, enhance the mechanical properties, and promote the reactive efficiency with Al. The chemical structure and molecular weight of copolyurethanes were characterized by attenuated total reflectance-Fourier transform-infrared spectroscopy (ATR-FTIR), nuclear magnetic resonance spectrometry (NMR), and gel permeation Table S1 chromatography (GPC). The impact sensitivity and mechanical properties of the copolyurethanes were tested by drop weight test, X-ray photoelectron spectroscopic (XPS), tensile test, and scanning electron microscopy (SEM), respectively. The thermal properties of copolyurethanes and their compatibility with Al and cyclotetramethylene tetranitramine (HMX) were also described by differential scanning calorimetry (DSC), thermal gravimetric analysis (TGA), and differential thermal analysis (DTA). The thermal performance of the PBFMO-*b*-GAP/Al complex was investigated by the cook-off test.

## 2. Experimental

### 2.1. Materials

GAP with molecular weight of 3500 g mol^−1^ and hydroxy value of 0.9% was provided from the Liming Chemical Engineering Research and Design Institute (Luoyang, China). Butane diol (BDO), BF_3_-etherate and dibutyltindilaurate (DBTDL) were purchased from J&K scientific Ltd. (Shanghai, China). Toluene diisocyanate (TDI), N,N-dimethylformamide (DMF), dichloromethane (DCM), and ethanol were supplied by Sinopharm Chemical Reagent Co., Ltd. (Xi’an, China). 1,2-dichloroethane was obtained from Jinshan Chemical Test Co., Ltd. (Chengdu, China). BDO and BF_3_-dimethyl ether were distilled under reduced pressure prior to use. All other solvents for the reactions were of analytical grade and distilled before use.

### 2.2. Polymerization of PBFMO

The PBFMO was synthesized via cationic ring-opening homopolymerization of [3,3-bis(2,2,2-trifluoroethoxymethyl) oxetane] glycol (BFMO), which was synthesized according to the literature procedure [25]. In brief, under a nitrogen atmosphere, BDO and BF_3_-etherate were dissolved in dried methylene chloride in a round bottom Schlenk flask and stirred for 1 h. BFMO was added into the mixture drop by drop over a period of 8 h, and the reaction mixture was then left under stirring for an additional 24 h. After the polymerization, sodium bicarbonate solution was added to terminate the reaction, and the organic phase was collected, washed by distilled water and dried over anhydrous Na_2_SO_4_ overnight. Finally, the solution was dried under 45 °C vacuum to yield a viscous wax PBFMO.

### 2.3. Synthesis of PBFMO-b-GAP Copolyurethanes

PBFMO-*b*-GAP copolyurethanes were synthesized through linking of GAP and PBFMO by TDI via a prepolymer process. In a typical synthesis example, GAP, PBFMO, and freshly distilled 1,2-dichloroethane were placed in a 250 mL four-neck flask equipped with a condenser, mechanical stirrer and thermometer under a nitrogen atmosphere, and then heated to 60 °C. TDI and DBTDL were then dissolved in 1,2-dichloroethane and added dropwise into the reaction solution. After stirring for an additional 2 h, the reaction mixture was poured into 400 mL ethanol. The polymer was precipitated and separated out and dried in vacuum at 50 °C for 24 h to obtain PBFMO-*b*-GAP copolyurethanes (yield, 97%).

### 2.4. Characterization

ART-FTIR spectra were recorded in a Bruker Tensor 27 spectrometer. ^1^H-NMR, ^13^C-NMR, and ^19^F-NMR were conducted on a 500 MHz Bruker spectrometer using deuterated chloroform as the solvent and tetramethylsilane as the internal standard. GPC was conducted on a Waters GPC, using tetrahydrofuran and polystyrene standards as the mobile phase and for calibration, respectively. The impact sensitivity was tested according to the national military standard GJB772-1997 method (the weight of dropping hammer 5 kg, and the drop height was between 0–1.29 m), and the characteristic drop height *H*_50_ (the drop hammer height of a statistical 50% probability of explosion) was taken to evaluate the impact sensitivity of samples. XPS analysis was performed using a Sigmaprobe instrument (ThermoElectron Corp., Pennsylvania, UK) equipped with a nonmonochromatic Al KR (hv =1486.6 eV) source at a power of 300 W. Mechanical test samples were prepared according to ASTM standard D-412 [26]. Mechanical properties of all the elastomers gels were measured on a Shimadzu AG-100kN X Plus universal testing machine (Shimadzu, Kyoto, Japan) in accordance with GB/T528–1998 with a tensile rate of 500 mm min^−1^ at 293 K. The dimensions of testing gels were 20 mm (neck length) × 4 mm (width) × 2 mm (thickness). Tensile tests were conducted five times independently, and results were presented as the mean in values ± standard deviation of quintuplicate measurements for the experiment. Morphology of the gels was investigated through SEM on a Tescan Vega 3 LMU scanning electron microscope (Tescan, Brnob, Czech Republic). All the elastomers gels were frozen in liquid nitrogen, snapped, and sputtered with gold until the experiment. DSC equipped with a TA instruments DSC Q1000 and TGA measurements of samples were performed in a nitrogen atmosphere using a SDT Q600 TGA instrument in the temperature range from 25 to 500 °C with heating rate of 10 K min^−1^.

**Figure 1:**
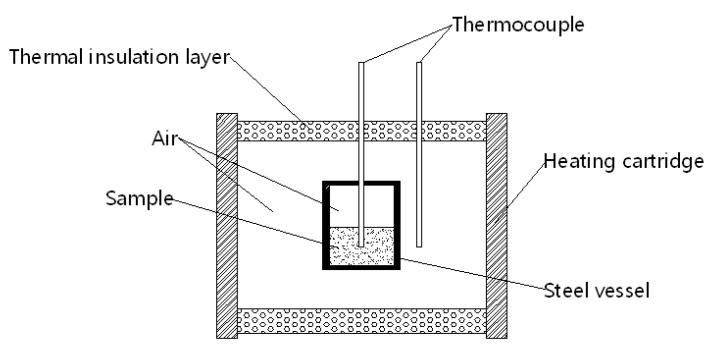
Schematic geometry of the cook-off test.

The special equipment for the slow cook-off test used in the research was designed by the Institute of Chemical Materials; its schematic diagram is shown in Figure 1. The equipment had a power of 1500 W, the heating rate of the heating cartridge wall was set at 1 °C min^−1^, and temperature ranged from room temperature to 250 °C. In the slow cook-off test, we put PBFMO-*b*-GAP/Al complex samples in the test set-up, heated by an electric heating cord; meanwhile, the equal heating components of PBFMO-*b*-GAP were heated with an intelligent temperature controller to adjust the heating rate. Samples were well sealed, and the thermocouples were utilized to obtain their temperature vs time curves. These experimental results were synthetically analyzed to investigate the thermal performance of the PBFMO-*b*-GAP/Al complex under slow heating stimulation.

## 3. Result and Discussion

### 3.1. Preparation of PBFMO-b-GAP Copolyurethanes

**Figure 2:**
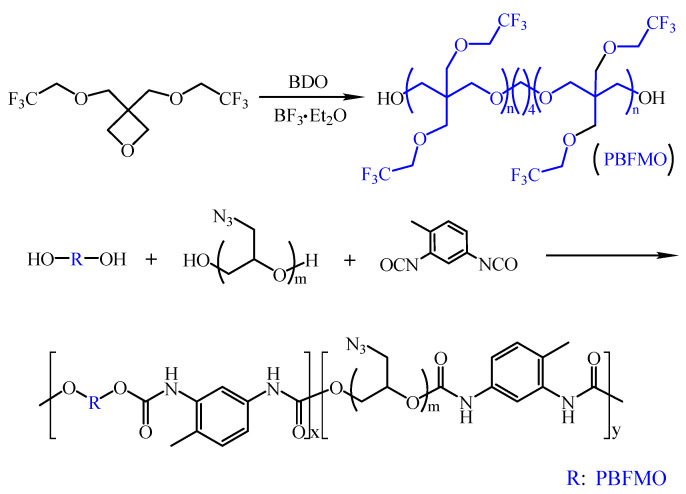
The synthesis route of PBFMO-*b*-GAP copolyurethanes.

**Figure 3:**
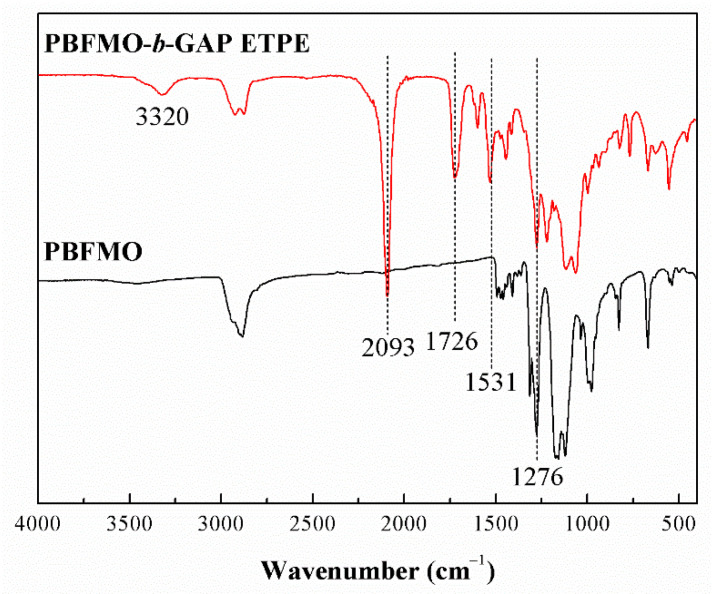
ART-FTIR spectra of PBFMO and PBFMO-*b*-GAP copolyurethanes.

The synthesis of PBFMO-*b*-GAP copolyurethanes was done via a prepolymer process, as illustrated in Figure 2. ART-FTIR and NMR were adopted to confirm the molecular structure of PBFMO-*b*-GAP. The peaks that appeared at 1135 and 1276 cm^−1^ in Figure 3 were due to the C–O–C and –CF_3_ stretching vibration of PBFMO, respectively [27,28]. The new stretching band at 2093 cm^−1^ was ascribed to the –N_3_ from GAP. The appearance of 3320 cm^−1^ was due to the –NH stretching vibration, the appearance of 1726 cm^−1^, 1531 cm^−1^ and 1376 cm^−1^ were assigned to C=O and –NH stretching bands of the urethane group [29,30]. These results confirmed the successful synthesis of the PBFMO-*b*-GAP polyurethane.

**Figure 4:**
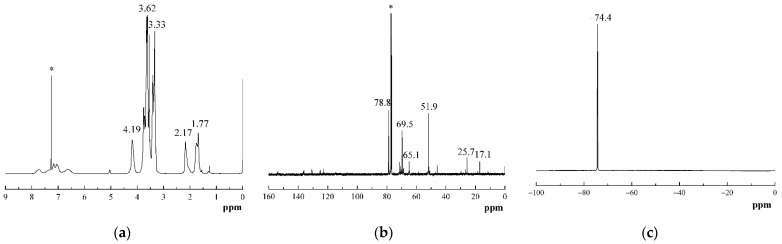
^1^H-NMR spectrum (**a**), ^13^C-NMR spectrum (**b**), and ^19^F-NMR spectrum (**c**) of PBFMO-*b*-GAP in CDCl_3_.

As shown in Figure 4a, proton signals due to methylene protons of GAP and PBFMO in the 3.62 ppm region as a broad band are clearly seen; methylene protons of the PBFMO side chain appeared in the 3.33 ppm region. The corresponding ^13^C-NMR (Figure 4b) also indicated the presence of all the carbons in GAP and PBFMO. The chemical shifts at 69.5 and 78.8 ppm are attributed to the carbons signals of methylene from GAP and PBFMO. A characteristic signal at 51.9 ppm due to the quaternary carbon atom of C–N_3_ was also observed [31]. The signals at 1.77 ppm and 2.17 ppm were assigned to the methyl protons of TDI and methylene protons of BDO, respectively, and the corresponding carbon signals appearing at 17.1 ppm and 25.7 ppm were also observed. Moreover, as shown in Figure 4c of ^19^F-NMR, the peak at -74.4 ppm was attributed to the –CF_3_ of the side chain [32]. These signal positions observed in the NMR spectra of PBFMO-*b*-GAP strictly corroborated the ART-FTIR results.

### 3.2. Density, Sensitivity, and XPS of PBFMO-b-GAP Copolyurethanes

To investigate the different PBFMO-*b*-GAP copolyurethanes, the molar ratio of PBFMO/GAP was set at 1/3, 1/9, and 1/19, during chain coupling by TDI to obtain PBFMO-*b*-GAP-1^#^, 2^#^ and 3^#^, respectively. As shown in Table 1, the number average molecular weight (*M*_n_) of PBFMO-*b*-GAP was around 3.0~3.3×10^4^ g mol^−1^, and its density was around 1.273–1.308 g cm^−3^, which was higher than that of the control group (GAP-based polyurethane, GAP-ETPE, 1.263 g cm^−3^). It is due to the polymers with –CF_3_ that the group side chain has a higher density [33]. 

Sensitivity of the energetic polymer is an important parameter because it is significantly connected with the safety operation and applications in the propellant [34]. In this study, the characteristic drop height *H*_50_ towards impact sensitivity was characterized by the drop weight test using standard procedures (listed in Table 1). It could be seen that the sensitivity of energetic materials was reduced as the mass ratio of polymer in the gel increased. Particularly noteworthy is the fact that the PBFMO/GAP molar ratio at 1/19 was markedly less sensitive than the pure GAP-based polyurethane. Therefore, it appeared that the introduction of fluoropolymer is manipulated to reduce the sensitivity of very high energy composite energetic materials made in this fashion, and it is easy to handle processability as propellant formulations.

**Figure 5:**
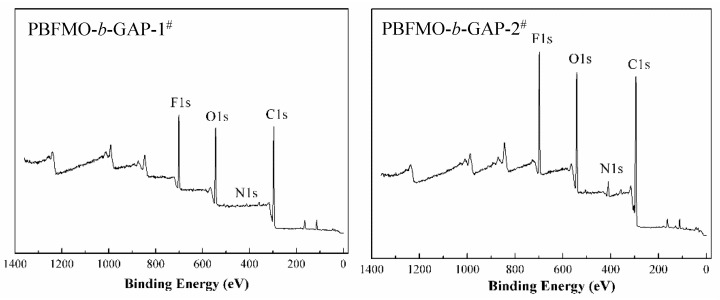

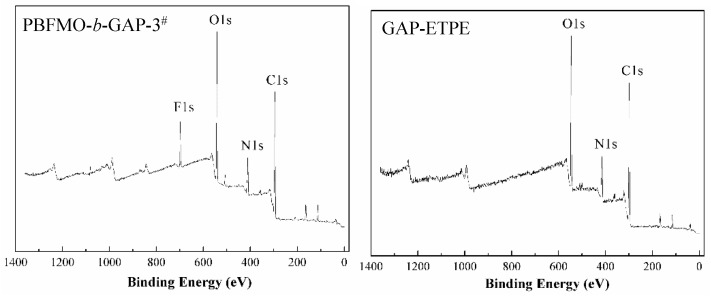
XPS curves of the gels prepared from GAP-based polyurethanes.

XPS was employed to detect the change in elementary composition on the elastomers surface, and provide valuable insight into the influence between sensitivity and fluorine content [35]. The N1s, C1s, O1s, and F1s elements of the XPS spectra of the elastomers surface and its surface compositions, expressed quantitatively as atomic weight percentages, were summarized in Figure 5 and Table 2, respectively. Due to the introduction of different content of PBFMO, the concentrations of F from the elastomers surface were 5.34%, 12.91%, and 13.05%; meanwhile, the theoretical contents of F elements were 2.02%, 4.04%, and 10.01%, respectively, and the atomic weight percentage of N obviously decreased from 12.16% to 1.54%. The values of F elements at the surface were much higher than its theoretical value, which was due to F atoms segregating on the surface and generating low wettability and coefficients of friction, as was also predicted in other literature [36,37]. The results indicate that the increase in F elements on the surface and the reduction of N elements create a satisfactory sensitivity of PBFMO-*b*-GAP.

### 3.3. Mechanical Properties of PBFMO-b-GAP Copolyurethanes

**Figure 6:**
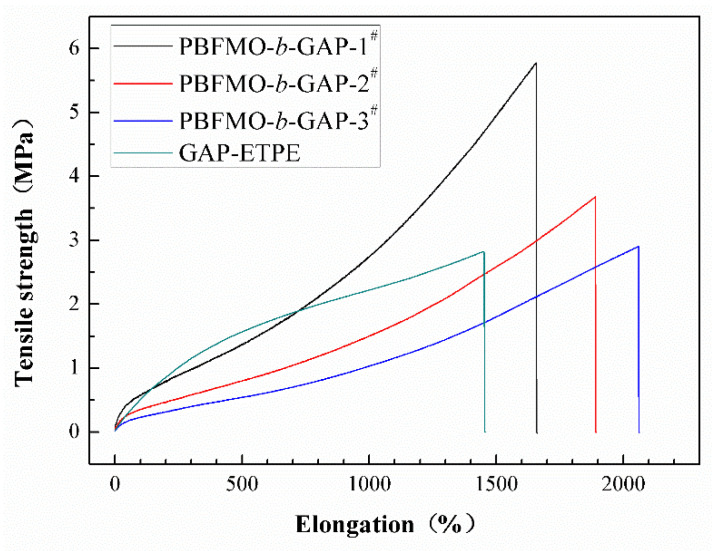
Mechanical properties of PBFMO-*b*-GAP copolyurethanes. (Tensile tests were conducted five times independently for each example, and the curve close to the average value was presented.)

Mechanical properties of the PBFMO-*b*-GAP copolyurethanes prepared from various ratio of PBFMO/GAP prepolymers were evaluated with a universal testing machine according to GB/T528-1998 (shown in Figure 6 and Table 3). It is clear that the tensile strength of PBFMO-*b*-GAP increased from 2.9 ± 0.11 to 5.75 ± 0.275 MPa, along with an increase in PBFMO content; meanwhile, the elongation at break decreased from 2056 ± 47.3% to 1660 ± 42.3%. The mechanical properties of PBFMO-*b*-GAP copolyurethanes were better than the reported literature, as shown in Table S1 [38,39]. In this work, PBFMO works as a hard segment in PBFMO-*b*-GAP copolyurethanes, and aggregates with each other to form physical cross-linking points. The rise in crosslinking density causes better tensile strength and lower breaking elongation. The results reveal that the PBFMO could be competent for hard segments in GAP-based polyurethanes to improve their mechanical properties.

**Figure 7:**
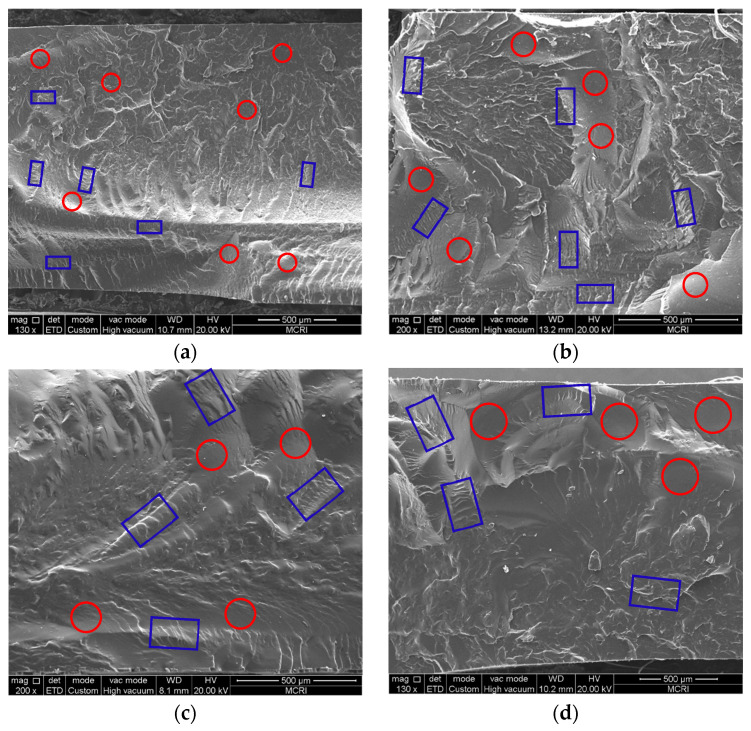
SEM images of the fracture surface of the gels prepared from (**a**) PBFMO-*b*-GAP-1^#^, (**b**) PBFMO-*b*-GAP-2^#^, (**c**) PBFMO-*b*-GAP-3^#^, and (**d**) GAP-ETPE. (wrinkles were marked as blue square frame, and ravines were marked as red circle frame).

The dispersion of hard segments and soft segments in PBFMO-*b*-GAP copolyurethanes is expected to affect their mechanical properties; SEM micrographs of freeze-fractured surfaces of the PBFMO-*b*-GAP copolyurethanes were displayed in Figure 7. The wrinkles (marked as blue square frame), representative of aggregated hard segments; and ravines (marked as red circle frame), representative of non-homogeneous soft segments, were observed in Figure 7. Generally, in ETPE, hard segments aggregate with each other to form physical cross-linking densities, and phase separation exists between the soft and hard segments. In this study, the dispersion of phase separation of hard and soft segments was obviously improved with an increase in PBFMO; meanwhile, the crosslinking density was enhanced, which was in correspondence with prior mechanical properties; the same phenomenon was found in other researches [40,41]. These results reveal that the introduction of PBFMO uniformly improved the phase separation of gels prepared from PBFMO-*b*-GAP, and resulted in good mechanical properties [42]. Considering PBFMO-*b*-GAP-1^#^ possessing the best tensile strength and sufficient elongation, it was selected in the next experiment.

### 3.4. Thermal Decomposition

**Figure 8:**
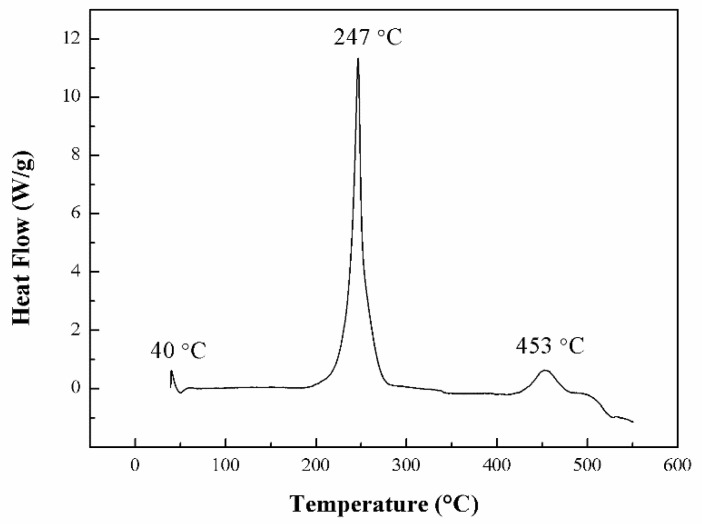
DSC curve of PBFMO-*b*-GAP copolyurethanes.

**Figure 9:**
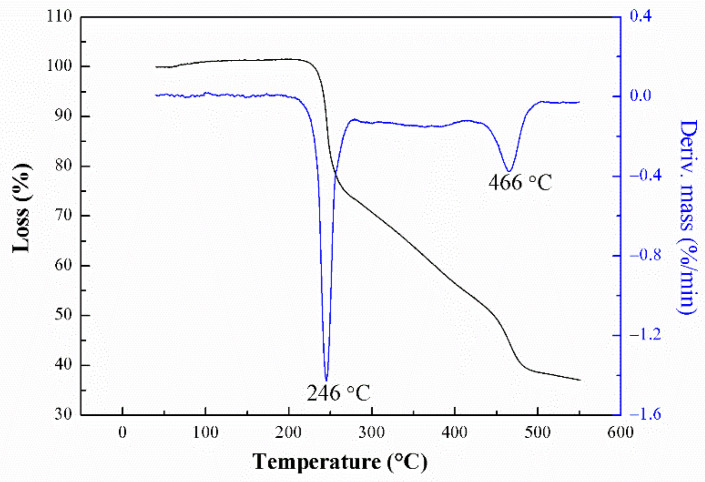
TG/DTA curves of PBFMO-*b*-GAP copolyurethanes.

It is well known that the thermal stability of energetic binders plays an important role in the preparation, processing, storage, and application of energetic materials [43,44]. Thus, DSC and TGA were used to study the thermal decomposition behavior of PBFMO-*b*-GAP copolyurethanes. The DSC curve of the PBFMO-*b*-GAP is presented in Figure 8, and the DSC curve of PBFMO-*b*-GAP showed three exothermic peaks. The first exothermic peak at 40 °C was the melting point of PBFMO, the second exothermic peak at 247 °C was caused by the decomposition of side chain azide groups on PBFMO-*b*-GAP to give nitrogen molecules, and the peak at 453 °C was due to PBFMO-*b*-GAP’s main chain decomposition [45]. The TGA and DTA traces of PBFMO-*b*-GAP are shown in Figure 9, displaying two distinct regions of weight loss. The first sharp weight loss of around 25% with respect to the total was at 246 °C, which corresponded to the stripping of the azide groups of the side chain, as described in DSC. After the first sharp step, the TGA curve showed a gradual weight loss, which corresponded to the incipient degradation of the main chain of PBFMO-*b*-GAP [46,47]. After the thermal decomposition period, the remaining residue was 35.9%.

### 3.5. Compatibility Testing

**Figure 10:**
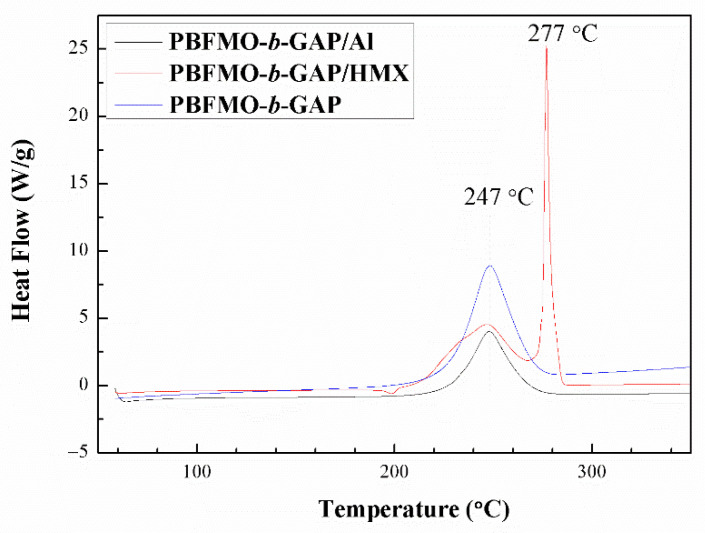
DSC curves of PBFMO-*b*-GAP, PBFMO-*b*-GAP/HMX complex, and PBFMO-*b*-GAP/Al complex.

Compatibility is an important safety and reliability index used to evaluate the production, application, and storage performance of energetic materials [48,49]. Usually, compatibility can be evaluated from DSC curves by studying the effect of the contact materials on the exothermic decomposition temperature of the explosives. In this study, DSC curves were used to determine the compatibility of PBFMO-*b*-GAP with the main energetic components, such as HMX and Al. Typical DSC curves of binary systems PBFMO-*b*-GAP/HMX, and PBFMO-*b*-GAP/Al are shown in Figure 10. According to the standards of compatibility, the binary systems PBFMO-*b*-GAP/HMX and PBFMO-*b*-GAP/Al had good compatibility because their Δ*Tp* values were all less than 2 °C [30]. This indicated that PBFMO-*b*-GAP could be safely used in HMX-based propellants.

### 3.6. Cook-Off Test

**Figure 11:**
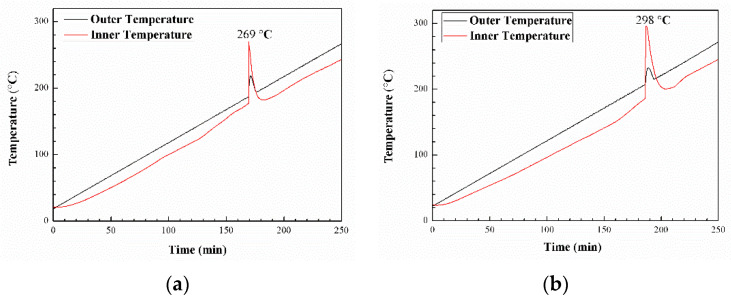
Cook-off curves of GAP-ETPE/Al (**a**) and PBFMO-*b*-GAP/Al (**b**).

The cook-off experiment was used to investigate the thermal performance of the PBFMO-*b*-GAP/Al complex, and the cook-off curves of PBFMO-*b*-GAP/Al composition are shown in Figure 11. Generally, the thermal decomposition characteristics of the PBFMO-*b*-GAP/Al complex are close-linked with the response temperature peak of cook-off curves [50,51,52]. In this study, the response temperature peak of PBFMO-*b*-GAP/Al composition was up to 298 °C, which was significantly higher than that of the control group (269 °C). It was due to the decomposition process of PBFMO-*b*-GAP releasing HF; subsequently HF could react with Al and give more exothermal reactions [53].

## 4. Conclusions

An energetic copolyurethane binder, PBFMO-*b*-GAP, was synthesized on GAP as a soft segment and TDI-extended PBFMO as a hard segment. From FT-IR, NMR, and GPC results, the PBFMO-*b*-GAP was synthesized successfully via a prepolymer process. The drop weight test and XPS results indicated that the introduction of fluoropolymer could evidently reduce the sensitivity of the PBFMO-*b*-GAP polyurethane. The PBFMO-*b*-GAP showed an enhanced tensile strength of 5.75 MPa with a breaking elongation of 1660%, and the tensile strength of PBFMO-*b*-GAP gels was enhanced with an increase in PBFMO content. The DSC and TGA/DTA curves indicated that PBFMO-*b*-GAP had adequate resistance to thermal decomposition up to 200 °C and began to decompose gradually at about 230 °C; it also had good compatibility with Al and HMX. The PBFMO-*b*-GAP/Al complex had a significant response temperature peak of 298 °C in the cook-off curves, which indicated that the PBFMO-*b*-GAP/Al complex has specific reaction energy.

## Figures and Tables

**Table 1:** Relative molecular mass, density, and *H*_50_ of PBFMO-*b*-GAP copolyurethanes in different molar ratios.

Sample	PBFMO/GAP Molar Ratio	Theoretical Content of F Element	*M*_n_ (10^3^ gmol^−1^)	Density (g cm^−3^)	*H*_50_ (cm)
PBFMO-*b*-GAP-1^#^	1/3	10.1	33	1.308	>129
PBFMO-*b*-GAP-2^#^	1/9	4.04	31	1.290	>129
PBFMO-*b*-GAP-3^#^	1/19	2.02	30	1.273	56.2
GAP-ETPE	0	0	32	1.263	9.55

**Table 2:** Atomic weight percentages of XPS curves.

Sample	C (%)	O (%)	N (%)	F (%)
PBFMO-*b*-GAP-1^#^	65.22	20.18	1.54	13.05
PBFMO-*b*-GAP-2^#^	63.00	19.76	4.33	12.91
PBFMO-*b*-GAP-3^#^	60.88	23.42	10.36	5.34
GAP-ETPE	63.8	24.04	12.16	0

**Table 3:** Mean values of PBFMO-*b*-GAP copolyurethanes in the tensile test.

Scheme	Tensile Strength (MPa)	Elongation at Break (%)
PBFMO-*b*-GAP-1^#^	5.75 ± 0.275	1660 ± 42.3
PBFMO-*b*-GAP-2^#^	3.65 ± 0.13	1874 ± 58
PBFMO-*b*-GAP-3^#^	2.9 ± 0.11	2056 ± 47.3
GAP-ETPE	2.81 ± 0.124	1446 ± 52

## Data Availability

The data presented in this study are available on request from the corresponding author.

## Supplementary Materials

The following are available online at https://www.mdpi.com/article/10.3390/polym13162706/s1, Table S1: Mechanical Properties of polymer film for SEM images in different literaures.
