# Supplementary material for: Fluoropolymer/Glycidyl Azide Polymer (GAP) Block Copolyurethane as New Energetic Binders: Synthesis, Mechanical Properties, and Thermal Performance
Source: Polymers (Basel). 2021 Aug 13;13(16):2706. doi: 10.3390/polym13162706 (PMC8399772; doi:10.3390/polym13162706)
Supplement: Supplementary file 1 [file polymers-13-02706-s001.zip › polymers-1311347-supplementary.pdf]

Supplementary Information

Table S1. Mechanical Properties of polymer film for SEM images in different literaures.

Samples	Raw materials	Stress (MPa)	Strain (%)	Dispersion of phase separation
PBFMO-*b*-GAP	GAP, PBFMO, TDI	5.75	1660	Uniformly dispersion
GAP-ETPE [1S]	GAP, N-(2-cyanoethyl) diethanolamine, HMDI	7.58	490	Nearly uniformity
poly(TFEE-r-GA) based polyurethane [40]	poly(TFEE-r-GA), TMP, IPDI	5.52	162.8	Relative uniformity
FGAP-based polyurethane [2S]	FGAP, Desmodur N-100	1.5	81.6	nonuniform

Supplementary References

1S. Z. Wang, T.F. Zhang, B.B. Zhao and Y.J. Luo, Effect of nitrocellulose (NC) on morphology, rheological and mechanical properties of glycidyl azide polymer based energetic thermoplastic elastomer/NC blends, Polym. Int. 66 (2017) 705-711.

2S. M.H. Xu, Z.X. Ge, X.M. Lu, H.C. Mo, Y.P. Ji and H.M. Hu, Structure and mechanical properties of fluorine-containing glycidyl azide polymer-based energetic binders, Polym. Int. 66 (9) (2017) 1318-1323.
